# Perioperative management in distal pancreatectomy: results of a survey in 23 European participating centres of the DISPACT trial and a review of literature

**DOI:** 10.1186/1745-6215-10-58

**Published:** 2009-07-26

**Authors:** Helge Bruns, Nuh N Rahbari, Thorsten Löffler, Markus K Diener, Christoph M Seiler, Matthias Glanemann, Giovanni Butturini, Christoph Schuhmacher, Inga Rossion, Markus W Büchler, Tido Junghans, the DISPACT Trial group

**Affiliations:** 1 Department of General, Visceral and Transplantation Surgery, Ruprecht-Karls-University Heidelberg, Im Neuenheimer Feld 110, 69120 Heidelberg, Germany; 2 Study Centre of the German Surgical Society (SDGC), Ruprecht-Karls-University Heidelberg, Im Neuenheimer Feld 110, 69120 Heidelberg, Germany; 3 Department of General, Visceral, and Transplantation Surgery, Universitätsmedizin Berlin, Augustenburger Platz 1, Charité Campus Virchow Klinikum, 13353 Berlin, Germany; 4 Policlinico Borgo Roma, Universita di Verona, Piazzale La Scuro 10, 37134 Verona, Italy; 5 Department of General Surgery, Technische Universität München, Ismaningerstrasse 22, Munich 81675, Germany; 6 Department of General, Visceral, Vascular and Thoracic Surgery, Universitätsmedizin Berlin, Charitéplatz 1, Charité Campus Mitte, 10117 Berlin, Germany

## Abstract

**Background:**

Concomitant treatment in addition to intervention may influence the primary outcome, especially in complex interventions such as surgical trials. Evidence-based standards for perioperative care after distal pancreatectomy, however, have been rarely defined. This study's objective was therefore to identify and analyse the current basis of evidence for perioperative management in distal pancreatectomy.

**Methods:**

A standardised questionnaire was sent to 23 European centres recruiting patients for a randomized controlled trial (RCT) on open distal pancreatectomy that would compare suture versus stapler closure of the pancreatic remnant (DISPACT trial, ISRCTN 18452029). Perioperative strategies (e.g., bowel preparation, pain management, administration of antibiotics, abdominal incision, drainages, nasogastric tubes, somatostatin, mobilisation and feeding regimens) were assessed. Moreover, a systematic literature search in the Medline database was performed and retrieved meta-analyses and RCTs were reviewed.

**Results:**

All 23 centres returned the questionnaire. Consensus for thoracic epidural catheters (TECs), pain treatment and transverse incisions was found, as well as strong consensus for the placement of intra-abdominal drainages and perioperative single-shot antibiotics. Also, there was consensus that bowel preparation, somatostatin application, postoperative nasogastric tubes and intravenous feeding might not be beneficial. The literature search identified 16 meta-analyses and 19 RCTs demonstrating that bowel preparation, somatostatin therapy and nasogastric tubes can be omitted. Early mobilisation, feeding and TECs seem to be beneficial for patients. The value of drainages remains unclear.

**Conclusion:**

Most perioperative standards within the centres participating in the DISPACT trial are in accordance with current available evidence. The need for drainages requires further investigation.

**Trial registration:**

***Clinical trial registration***: ISRCTN 18452029

## Background

Short-term outcomes after major abdominal surgery are influenced by indication, surgical intervention, the surgeons' expertise, hospital volume and perioperative management [1-5]. In order to qualitatively assess the impact of the various factors on outcome, a rigorous means of evaluation such as the randomized controlled trial (RCT) design is necessary. Mortality and morbidity in pancreatic surgery has decreased in high-volume centres in recent years [6,7] mainly by virtue of standardised surgical and optimised perioperative management [8]. In high volume centres, mortality from distal pancreatectomy is as low as 5% while morbidity remains as high as 40% [7,9-12]. Morbidity is determined by perioperative factors such as bowel preparation, incision type, analgesia, mobilisation, and feeding regimens, amongst other factors [1]. In addition, long-term survival after pancreatectomy as cancer treatment strongly depends on a centre's operative volume [13].

A recent study of 617 consecutive pancreatic resections demonstrated local complications to be more frequent than systemic complications [14]. Thus, the formation of pancreatic fistulas can be considered the most important complication after a pancreatic left resection. Currently, the Study Centre of the German Surgical Society is conducting a large RCT (DISPACT trial, ISRCTN 18452029) to compare the occurrence rates of pancreatic fistula after stapler versus hand-sewn closure of the pancreas after left resections [15]. Both expert opinions and evidence-based standards were taken into account in the development of the study protocol. The consensus-assisted development of study protocols has proven to be beneficial and may indeed be helpful in identifying when consensus is present but not justified by evidence [16]. All 23 centres contributing to DISPACT are centres with a substantial caseload of pancreatic left resection (Table 1). The study protocol of DISPACT has been approved by the ethics committees of all participating centres and has been recently published [15]. Since the DISPACT trial aims to evaluate the effect of two surgical procedures (stapler and hand-sewn closure) on the development of pancreatic fistula, surgical and perioperative standardisation is of special interest. The present study was launched during an investigator meeting to identify the current practices concerning perioperative management amongst the various participating centres. This study aims to identify the level of consensus of relevant factors in perioperative management. In addition, an extensive literature search was conducted to compare findings with current best available evidence.

**Table 1:** Total pancreatectomies and distal pancreatic resections performed in 2008 at centres taking part in DISPACT Trial

Centre	Number of pancreatic resections	Number of distal pancreatectomies	Number of patients randomized in 2008
Amsterdam, Netherlands	76	12	8
Berlin Charité Mitte, Germany	20	12	4
Berlin Charité Virchow, Germany	197	48	20
Berlin Lichtenberg, Germany	38	10	4
Bochum St. Josef, Germany	214	46	7
Dresden-Friedrichstadt, Germany	52	5	2
Freiburg, Germany	83	15	2
Gent, Belgium	68	6	3
Heidelberg, Germany	423	87	45
Homburg, Germany	61	10	-
Cologne-Merheim, Germany	25	10	-
Liverpool, Great Britain	128	13	1
Ljubljana, Slovenia	90	25	10
Mannheim, Germany	71	10	4
Marburg, Germany	48	13	1
Munich-LMU, Germany	91	19	13
Munich-TU, Germany	87	27	22
Regensburg, Germany	70	19	6
Verona, Italy	269	66	17
Würzburg, Germany	20	7	2
Wuppertal, Germany	24	3	1
Total	2155	463	172
Median (range)	71 (20–423)	13 (3–87)	4 (0–45)

## Methods

### Survey of current practice

Twenty-three European centres participating in the DISPACT trial were evaluated using a standardized questionnaire to evaluate perioperative management [see Additional file 1]. Standards in bowel preparation, pain and feeding management, antibiotics prophylaxis, incision type, drainages, necessity and duration of intensive care, mobilisation and perioperative somatostatin therapy were assessed. These relevant items were identified in a previously published meta-analysis and included in the questionnaire [17]. The survey was designed by T.L., N.N.R. and C.M.S and mailed to all centres taking part in the DISPACT trial. The survey was completed and returned by all local surgical sub-investigators. Consensus levels were classified for each outcome as published elsewhere [18]. Briefly, a discrete scale was used and the results were stratified along consensus levels 1 – 4, which in turn corresponded to being standard in <50% (no consensus), 50–75% (overall agreement), 75–95% (consensus), and >95% (strong consensus) of participating centres, respectively.

For time-critical factors such as the removal of intra-abdominal drainages, thoracic epidural catheters (TECs) and gastric tubes; the discharge from intensive care unit (ICU) or intermediate care (IMC) wards; and the duration of intravenous feeding and somatostatin therapy, median time and interquartile ranges (IQRs) have been calculated.

### Literature search of best practice

Only RCTs or meta-analyses of RCTs assessing perioperative care were eligible for inclusion. Since specific evidence of perioperative care for distal pancreatectomy and pancreatic surgery was expected to be low, articles on general abdominal surgery including hepatic and colorectal surgery were included as well and evaluated in hierarchical order (distal pancreatectomy > pancreatic surgery > abdominal surgery > hepatic and colorectal surgery). Studies of paediatric, laparoscopic, and transplantation surgery were excluded. A search algorithm was developed and an extensive systematic Medline search using Boolean operator functions and wildcards (i.e. the asterisk symbol) was performed independently by three authors of this study (H.B., N.N.R. and T.L.). The search algorithm used single relevant keywords, medical subject headings (MESH), and their combinations (Appendix 1). No other literature databases than Medline were searched and a subsequent analysis of the identified literature was performed. All published articles were evaluated.

Reference lists of the retrieved literature were manually cross-searched for additional publications independently by three researchers (T.L., N.N.R and H.B.) under the supervision of an experienced researcher (C.M.S.). Moreover, captured citations were filtered for study design in order to identify all RCTs and meta-analyses. Titles, abstracts, and full text articles were screened for the selection of relevant studies.

## Results

### Survey of current practice

All 23 centres participating in the DISPACT trial returned completely filled out questionnaires either per fax, mail or email. The annual caseload of pancreatic resections for each centre within the year 2008 is given in Table 1. All centres performed at least 20 pancreatic resections, which was the requirement for participation in the DISPACT trial. Pancreatic left resections (median 13; range 3–87) are performed less commonly than other resections. Only two centres have not randomized patients for DISPACT within the last year. Median 4 patients (range 0–45) were randomized. The random ratio (randomized left resections/total left resection * 100) was 37%.

#### Bowel preparation

Mechanical bowel preparation (MBP) as a standard was performed at eight of the 23 participating centres (Table 2). Enemas were made use of at seven centres and one centre performed orthograde lavage; 14 hospitals did not use any kind of MBP before left pancreatectomy (Table 2).

**Table 2:** Perioperative standards in the 23 European centres for pancreatic surgery

	n	%	consensus level	median duration (IQR)
Bowel preparation				
none	15	65	overall agreement	-
enema	7	30		-
orthograde lavage	1	4		-
Type of incision				
Midline^+^	5	22		-
Transverse^+^	18	78	consensus	-
Other	2	4		-
Intraoperative single-shot antibiotic prophylaxis				
No	1	4		-
Yes	22	96	strong consensus	-
Intra-abdominal drainages				
No	0	0		
Yes	23	100	strong consensus	4 (3–5)
Postoperative care				
IMC**	9	39		2 (1–2.25)
ICU**	11	48		1 (1-1)
Nursery ward	5	22		-
Not specified	1	4		-
Pain management (thoracic epidural catheter)				
No	3	13		-
Yes*	20	87	consensus	4 (3–5)
Post-operative gastric tube				
No	13	57	overall agreement	-
Yes	10	43		1 (1–2)
Intravenous feeding				
No	12	52	overall agreement	-
Yes	11	48		2 (1.5–2)
Somatostatin therapy				
No	15	65	overall agreement	-
Yes	8	35		6 (3.75–7)

^+ ^One centre reported using both midline and transversal incisions frequently.

*Local anaesthetics and opioids were used at 75% of the centres (n = 15), 15% (n = 3) used local anaesthetics without opioids, and 10% (n = 2) did not specify administered drugs.

** Patients were transferred from the ICU to the IMC at three centres. While there is no consensus whether patients should be transferred to the ICU or IMC, there is an overall agreement in 74% of centres (n = 17) that patients should not be returned to nursery wards immediately after distal pancreatectomy.

#### Type of incision, intraoperative antibiotics and placement of abdominal drainages

The transverse incision was exclusively used by 16 hospitals and the midline by four hospitals. Two centres used other types of incisions and one centre used both incisions, depending on the individual surgeon's choice (Table 2). Intraoperative single-shot antibiotics were performed in all but one centre (Table 2).

All centres placed intra-abdominal drainages. Drains were left for median of four days (IQR 3–5), with a maximum of eight days at one centre (Table 2).

#### Postoperative care

Postoperatively, 17 centres transferred their patients to ICU or IMC wards, whereas five centres transferred patients directly to nursery wards. Patients were discharged from ICU and IMC after a median of one day (IQR 1-1) and two days (IQR 1–2.25), respectively (Table 2).

#### Pain management

Thoracic epidural catheters (TECs) were used in 20 hospitals and kept in place for a median duration of four days (IQR 3–5) (Appendix 1). Fifteen centres used a combination of local anaesthetics and opioids for the medication of TECs and three centres used exclusively local anaesthetics (Appendix 1). Two centres did not specify the medication.

#### Nasogastric tube and feeding regimens

Postoperatively, a nasogastric tube was placed for a median of one day (IQR 1–2) in 10 hospitals (Table 2). Eleven centres used parenteral feeding for a median of two days (IQR 1.5–2) (Table 2). Oral feeding with fluids started on day 1 (IQR (0–1) and solid food on day 2 (IQR 2–3) (Table 3). One centre reported keeping patients on parenteral feeding till day 5 and starting oral feeding with fluids and solids on day 5.

**Table 3:** Mobilisation and oral feeding

Begin of...	postoperative day (median (IQR))
...mobilisation	1 (0–1)
...oral feeding	
Fluids	1 (0–1)
Solid food	2 (2–3)

#### Mobilisation

Mobilisation of patients started at postoperative day 1 (IQR 0–1) (Table 3). Eight centres started mobilisation on the operative day, while 14 centres started on first postoperative day. One centre reported starting mobilisation on the second postoperative day.

#### Somatostatin therapy

Fifteen centres did not use Somatostatin as a routine after pancreatic surgery. In eight centres, somatostatin was used as a standard after left pancreatectomy for a median of six days (IQR 3.75–7) (Appendix 1).

### Literature search of best practice

All of the identified studies focus on large bowel surgery. Besides pancreatic surgery, other procedures have been included based on the similarity in concepts of perioperative management and determinants of complications.

A total of 16 meta-analyses of RCTs and 19 RCTs were identified and reviewed [see Additional file 2].

#### Bowel preparation

We identified two meta-analyses [19,20] which included 16 RCTs and six RCTs [21-26] with a total number of 3046 patients and 3747 patients, respectively [see Additional file 2]. All of these studies failed to identify a benefit from bowel preparation; both meta-analyses [19,20] and two of the six RCTs [21,25] found beneficial effects of no bowel preparation in terms of decreased anastomotic leakage and/or wound infections. Slim et al. [19] and Guenaga et al. [20] identified a rate of 6.3% vs. 3.2% (p = 0.003) and 5.6% vs. 3.2% (p = 0.032) for bowel preparation versus no bowel preparation, respectively [see Additional file 2].

#### Type of incision

While complication rates were not different in the meta-analysis by Brown et al. [27] in transversal versus midline incisions, a recent RCT by Fassiadis et al. [28] identified a decreased risk of incisional hernia after transverse incision for abdominal aortic aneurysm repair only (p = 0.01; [see Additional file 2]. Moreover, Brown et al. [27] suggested that transverse incisions may be less painful (odds ratio [OR] for less analgesic use (mg morphine equivalent) for total hospital stay, -6.29; 95% CI [-12.57; -0.01]) and may affect pulmonary function less than midline access (OR for percentage change in forced expiratory volume in one second on the last day of measurement, 18.31; 95% CI [6.84; 29.78]).

#### Perioperative antibiotic prophylaxis

Regarding antibiotic prophylaxis, the meta-analysis by Song et al. [29], which included 147 RCTs, showed single-shot antibiotics to be as effective as long-term antibiotics in the prevention of surgical wound infections [see Additional file 2]. The recent study by Fujita et al. [30] identified a dose-dependent correlation between surgical site infections and antibiotics (p = 0.009; [see Additional file 2].

#### Abdominal drainages

Gurusamy et al. [31] included five trials and 465 patients in their meta-analysis [see Additional file 2]. No significant influence of drainages on complication rates after liver resection was found [see Additional file 2] [31]. Petrowsky et al. [32] were able to identify surgical procedures after which abdominal drainages significantly decrease complications (i.e. oesophageal resection and total gastrectomy) and in which abdominal drainages can be omitted (i.e. hepatic, rectal or colonic resection with primary anastomosis, and appendectomy) [see Additional file 2]. In a RCT by Conlon et al. [33], no correlation between the placement of abdominal drainages and the need for interventional drainages and surgical exploration after septic complications in patients undergoing pancreatectomy was detected [see Additional file 2]. For hepatectomy, Sun et al. [34] were able to identify a correlation of abdominal drainages and postoperative wound complications in a RCT (28% vs. 3%, p < 0.001; [see Additional file 2].

#### Pain management

In their meta-analysis of 16 RCTs, Marret et al. [35] did not identify any differences in length of hospitalisation between patients receiving epidural or parenteral analgesia after colorectal surgery [see Additional file 2]. Werawatganon et al. [36], who included 711 patients from nine RCTs in their analysis, and Wu et al. [37] identified better pain relief with epidural analgesia compared to intravenous analgesia [see Additional file 2]. In a meta-analysis by Jørgensen et al. [38], a decreased rate of gastrointestinal paralysis with comparable effect on pain was found for epidural local anaesthetics compared to opioid-based medication [see Additional file 2]. In RCTs, Flisberg et al. [39] and Mann et al. [40] were able to identify better pain relief with epidural analgesia and less frequent side effects compared to i.v. analgesia during rest (p < 0.003) and mobilisation (p < 0.001) [39] and rest (p < 0.001) and coughing (p < 0.002) [40], respectively [see Additional file 2].

#### Nasogastric tubes

Two meta-analyses by Nelson et al. [41,42] did not identify any advantage of routinely placed gastric tubes after abdominal surgery [see Additional file 2]. Additionally, an earlier return of bowel function was found in patients without gastric tube in both studies (p < 0.001) [41,42] [see Additional file 2]. Pesseaux et al. [43] included 200 patients after hepatic resection in a RCT and did not find an advantage for nasogastric tubes. They demonstrated an increased risk of pulmonary complications (i.e. pneumonia and atelectasis, p = 0.047 and p = 0.043, respectively) within the nasogastric tube group [see Additional file 2] [43].

#### Feeding regimens

Andersen et al. [44] included 13 RCTs with 1173 patients in their meta-analysis. No advantage for late return to oral diet was found [see Additional file 2] [44]. Han-Geurts et al. [45], who included 128 patients in a RCT on early (i.e. within the first two postoperative days) versus late (i.e. more than two days after procedure) postoperative return to oral diet, also failed to identify a significant difference in or beneficial effect of keeping patients starved in terms of postoperative ileus and recovery [see Additional file 2].

#### Mobilisation

Three RCTs [46-48] on postoperative mobilisation and physiotherapy were available [see Additional file 2]. While Browning et al. [46] concluded that early mobilisation may decrease hospitalisation length, Mackay et al. [48] did not find a benefit of chest physiotherapy in high risk patients after open abdominal surgery as far as pulmonary complications are concerned [see Additional file 2]. In the larger study by Fagevik Olsén et al. [47], chest physiotherapy decreased postoperative pulmonary complications and improved the mobilisation of patients (6% in physiotherapy group vs. 29% in control group; p < 0.001; [see Additional file 2].

#### Somatostatin therapy

While Connor et al. [49] and Alghamdi et al. [50] identified a reduced complication rate in their meta-analyses on somatostatin versus no somatostatin after pancreatic surgery (p = 0.003 [49] and p = 0.004 [50]), Zeng et al. [51] did not find any significant effect on morbidity [see Additional file 2]. All included meta-analyses [49-51] failed to identify any effect of somatostatin on mortality rates [see Additional file 2].

No beneficial effect of somatostatin was found in all included RCTs [52,53] on somatostatin after pancreatic surgery [see Additional file 2]. Hesse et al. [52] did not identify any reduced rate of pancreatic fistula. Even worse, Shan et al. [53] in their study found delayed gastric emptying to be more frequent in the somatostatin group.

## Discussion

Surgical and perioperative management remains a matter of debate. Besides surgical expertise and technique [9,13], perioperative procedures significantly influence morbidity [1,6]. This survey of 23 centres participating in a large multicenter trial on pancreatic left resection revealed that general perioperative standards can be detected in pancreatic surgery. In most items, at least overall agreement was present. Strong consensus was found in two items (i.e. placement of abdominal drainages and intraoperative antibiotic prophylaxis) and consensus was present for the usage of TECs for pain medication and transversal incisions as the abdominal cavity opening (Table 2). The DISPACT centres agreed overall that no bowel preparation, gastric tube, somatostatin therapy or intravenous feeding was necessary after left pancreatectomy (Table 2).

While most of the strategies and standards in the DISPACT group are in accordance with current evidence, this survey has detected some differences in the perioperative management after pancreatic left resection despite current available evidence from meta-analyses and RCTs.

### Bowel preparation

As early as 1972, Hughes [54] has questioned the beneficial effect of bowel preparation in large-bowel surgery. However, this tradition has remained a standard in abdominal surgery for decades. Within the DISPACT group, there was overall agreement that no bowel preparation is necessary before pancreatic left resection (Appendix 1). Based on the results from our literature research, this standard is in accordance with current evidence. Recent meta-analyses [19,20] and RCTs [21-26] did not identify any benefit from bowel preparation before abdominal surgery (Table 3A). In both meta-analyses by Guenaga et al. and by Slim et al. [19,20], an increased rate of anastomotic leakage was identified. Moreover, bowel preparation may even be disadvantageous in terms of wound infections [19-21].

In eight (35%) of 23 DISPACT centres, bowel preparation was used as a standard prior to left pancreatectomy. This is in contrast to the results of studies conducted in colorectal surgery demonstrating no significant differences for anastomotic leakage rates and overall morbidity [see Additional file 2] [19-26]. While there is no evidence that enema or orthograde lavage may be harmful in pancreatic surgery, it seems likely that there is no benefit from bowel preparation. Therefore, bowel preparation can no longer be considered as a necessary standard in pancreatic surgery. Most DISPACT centres have therefore already stopped any bowel preparation.

### Type of incision

A recent meta-analysis did not show any significant difference between midline and transverse incision as far as complication rates, incisional hernias and recovery times [see Additional file 2] [27,28]. Amongst the DISPACT centres, there was consensus (78%) that a transverse incision may be more beneficial for distal pancreatectomy (Table 2). While the meta-analysis by Brown et al. [27] failed to identify any difference between groups with midline and transverse abdominal incisions, Fassiadis et al. [28] found increased rates of incisional hernia in patients undergoing surgery for aortic aneurysms after midline versus transversal incision (20 of 22 patients versus 6 of 15 patients, respectively). This finding is confounded by the underlying connective tissue disorders which is a causative agent for both diseases and therefore may not be relevant for benign and malignant pancreatic disorders [55-57]. Therefore, the practise of the DISPACT centres is in accordance with the evidence.

### Antibiotic prophylaxis

Antibiotic prophylaxis significantly reduces surgical site infections as long as local tissue concentrations reach an effective level [see Additional file 2] [29,30]. Both single-shot and long-term antibiotics are equally effective. As a result, antibiotic prophylaxis is a common procedure in surgery.

All but one centre agreed on using single-shot antibiotics before left pancreatectomy (Appendix 1). Given the ubiquitous use of antibiotic prophylaxis in surgery, it can be suspected that one centre chose a different regime and may have used a long-term antibiotic prophylaxis.

### Abdominal drainages

Placement of drainages has a long tradition in abdominal surgery and can be dated back to the 19^th ^century. While many surgical procedures originating from these times have been omitted, Lawson Taits advice of "when in doubt, drain" is still considered standard in many surgical centres [58]. Not surprisingly, all centres taking part in the DISPACT trial agreed on using intra-abdominal drainages as a standard after distal pancreatectomy (Table 2). There were considerable differences in time concerning the removal of drainages, though; the median day of removal was the fourth postoperative day (IQR 3–5). Some centres kept drainages as a standard for up to eight days.

The detection of postoperative pancreatic fistulas according to the consensus definition requires a drainage for at least three days [59]. Since the occurrence of pancreatic fistula is the primary endpoint of the DISPACT trial, placement of abdominal drainages had to be included as a standard in the study protocol. Unfortunately, there is evidence that intra-abdominal drainages are not beneficial in all cases and sometimes may even be harmful [see Additional file 2] [31-34]. For a number of procedures (i.e. liver resection, appendectomy and colonic or rectal resection with primary anastomosis), there is no need for abdominal drainages while for other procedures (i.e. oesophageal resection or total gastrectomy) there are proven benefits [32]. Moreover, wound complications may even be more common when drainages are placed [34].

A recent RCT by Conlon et al. [33] failed to show a decreased mortality or rate of complications by intraperitoneal drainages after pancreatectomy [see Additional file 2]. A total number of 179 patients were included. Forty of them underwent distal pancreatectomy; data was consistent among the different procedures in pancreatic surgery. No reduced need for interventional drainages and surgical exploration for septic complications was found in the drainage group [33].

In summary, the value of drainages in pancreatic surgery remains unclear. Further studies are necessary to resolve the currents uncertainties regarding drainages after pancreatic surgery.

### Postoperative care

Most centres (74%) agree that patients undergoing distal pancreatectomy should be transferred to ICU or IMC wards (Table 2). In 22% of the DISPACT centres, patients are transferred directly to regular nursery wards. No meta-analyses or RCTs were identified in our Medline research regarding this topic.

### Pain management

Pain management is generally considered a key factor of fast track concepts in surgery and a determinant of hospitalisation length [60,61]. In 20 (87%) of the DISPACT centres, a thoracic epidural catheter was used for pain management. Fifteen centres (75%) applied regimens consisting of local anaesthetics and opioids (Table 2). While overall time of hospitalisation is not significantly different between patients receiving epidural and parenteral opioids, epidural administration significantly decreases side effects [see Additional file 2] [35-37,39,40]. In RCTs published by Filsberg et al. [39] and Mann et al. [40], epidural versus intravenous application of pain medication was compared [see Additional file 2]. Opioid-related gastrointestinal side effects especially are less frequent in epidural analgesia groups [39,40]. In the study by Jørgensen et al. [38], no difference in pain relief could be detected between opioid-based analgesia and analgesia using epidural application of local anaesthetics only. Within the DISPACT group, thoracic epidural catheters (TEC) were clearly preferred for pain management and placed for median of four days (IQR 3-3, Table 2). The preference for opioids in this context seems to be questionable.

### Nasogastric tubes

Most DISPACT centres do not apply post-operative gastric tubes as a standard after distal pancreatectomy (Table 2). Ten centres (43%) keep nasogastric tubes in place for a median of one day (IQR 1–2) (Table 2). Both meta-analyses and RCTs with a total number of 9634 included patients could not detect benefits from nasogastric tubes after abdominal surgery [see Additional file 2] [41-43]. Contrary to traditional belief, gastric tubes as a postoperative standard seem to be associated with a higher risk of pulmonary complications and delayed return of bowel function [see Additional file 2] [41-43]. Postoperative removal of nasogastric tubes is a standard based on evidence.

### Feeding regimens

Administration of oral fluids was performed on median postoperative day 1 (IQR 0–1) and of solid food on day 2 (IQR 2–3) (Table 3). Most centres did not perform intravenous feeding (Table 2). Both the RCT by Han-Geurts et al. [45] and the meta-analyses by Andersen et al. [44] did not find any benefit in keeping patients starved after abdominal surgery [see Additional file 2]. Since an early (i.e. second postoperative day) return to an oral diet is not associated with increased postoperative complications, there is no rationale in keeping patients starved [see Additional file 2] [44,45].

### Mobilisation

Early postoperative patient mobilisation (median postoperative day 1 (IQR 0–1) is performed in most of the centres (Table 3)). While early mobilisation actually may reduce length of hospitalisation, the value of postoperative chest physiotherapy remains unclear [see Additional file 2] [46-48]. Mackay et al. [48] could not detect any significant decrease in pulmonary complications with chest physiotherapy in high risk patients. Contrary to this, Fagevik et al. [47] reported a significant decrease in postoperative pulmonary complications and an improved mobilisation with chest physiotherapy [see Additional file 2].

### Somatostatin therapy

Fifteen (65%) DISPACT centres do not use somatostatin as a postoperative standard (Appendix 1). All reviewed studies agree that prophylactic somatostatin does not decrease mortality [see Additional file 2] [49-53,62]. As far as meta-analyses are concerned, the effect of somatostatin on the occurrence of pancreatic fistula remains unclear [49-51]. All reviewed RCTs not only agree that somatostatin does not have any influence on occurrence of pancreatic fistula, but Shan et al. [53] also found increased rates of delayed gastric emptying after prophylactic somatostatin administration and provided decreased plasma motilin levels as a possible mechanism [see Additional file 2] [52,62]. Therefore, somatostatin as a postoperative standard should be avoided.

## Conclusion

Evidence-based perioperative management is an important cornerstone for a successful outcome after complex surgical procedures. The results of this survey and the comparison with the current available evidence detected some inconsistencies in perioperative management of patients with pancreatic left resection. At least overall agreement was present in most items and was – with the exception of abdominal drainages – in accordance with strategies that were identified as beneficial for the patients from our literature research. Evidence-based perioperative treatment in pancreatic surgery requires the further conduct of randomized controlled trials.

## Abbreviations

DISPACT: DIStal PAnCreaTectomy; ICU: Intensive Care Unit; IMC: InterMediate Care; IQR: Inter Quartile Range; ISRCTN: International Standard Randomized Controlled Trial Number; MBP: Mechanical Bowel Preparation; MESH: Medical Subject Headings; RCT: Randomized Controlled Trial; TEC: Thoracic Epidural Catheter.

## Competing interests

The authors declare that they have no competing interests.

## Authors' contributions

HB performed the analysis, literature research and drafted the manuscript. NRR and TL performed the survey and literature search. MKD and TJ designed the study. CMS designed the study and reviewed the manuscript. MG, GB, CS, IR and MWB reviewed and contributed substantially to the manuscript. All authors read and approved the final manuscript.

## Appendix 1

### Items and corresponding search algorithms used for Medline search of literature-based standards

#### Bowel preparation

(Pancreas surgery [tw] OR pancreas resection [tw] OR pancreatic surgery [tw] OR pancreatic resection [tw] OR distal pancreatectomy [tw] OR pancreatic left resection [tw] OR pancreaticoduodenectomy [tw] OR hepatic surgery [tw] OR hepatic resection [tw] OR liver surgery [tw] OR hepatectomy [tw] OR colorectal surgery [tw] OR colon resection [tw] OR colon surgery [tw] OR abdominal surgery [tw] OR gastrointestinal surgery [tw]) AND (Mechanical bowel preparation [tw] OR bowel preparation [tw] OR bowel cleansing [tw] OR colon preparation [tw]) AND (randomized controlled trial [pt] OR controlled clinical trial [pt] OR randomized controlled trials [MESH] OR random allocation [MESH] OR double-blind method [MESH] OR single-blind method [MESH] OR clinical trial [pt] OR clinical trials [MESH] OR ("clinical trial" [tw]) OR ((singl* [tw] OR doubl* [tw] OR tripl* [tw]) AND (mask* [tw] OR blind* [tw])) OR placebos [MESH] OR placebo* [tw] OR random* [tw] OR research design [mh:noexp] OR Meta-Analysis [ptyp] OR systematic review [tw] NOT (animal [MESH] NOT human [MESH]) NOT case reports [pt] NOT Comment [PT] NOT letter [PT] NOT child [MESH] NOT infant [MESH] NOT child [MESH] NOT infant [MESH])

#### Thoracic epidural catheter

((Pancreas surgery [tw] OR pancreas resection [tw] OR pancreatic surgery [tw] OR pancreatic resection [tw] OR distal pancreatectomy [tw] OR pancreatic left resection [tw] OR pancreaticoduodenectomy [tw] OR hepatic surgery [tw] OR hepatic resection [tw] OR liver surgery [tw] OR hepatectomy [tw] OR colorectal surgery [tw] OR colon resection [tw] OR colon surgery [tw] OR abdominal surgery [tw] OR gastrointestinal surgery [tw]) AND (Thoracic epidural catheter [tw] OR epidural catheter* [tw] OR epidural analgesia [MESH] OR epidural opioid [tw] OR epidural local anaesthetic [tw]) AND (randomized controlled trial [pt] OR controlled clinical trial [pt] OR randomized controlled trials [MESH] OR random allocation [MESH] OR double-blind method [MESH] OR single-blind method [MESH] OR clinical trial [pt] OR clinical trials [MESH] OR ("clinical trial" [tw]) OR ((singl* [tw] OR doubl* [tw] OR tripl* [tw]) AND (mask* [tw] OR blind* [tw])) OR placebos [MESH] OR placebo* [tw] OR random* [tw] OR research design [mh:noexp] OR Meta-Analysis [ptyp] OR systematic review [tw] NOT (animal [MESH] NOT human [MESH]) NOT case reports [pt] NOT Comment [PT] NOT letter [PT] NOT child [MESH] NOT infant [MESH])

#### Somatostatin

(Gastrointestinal surgery [tw] OR pancreas surgery [tw] OR pancreas resection [tw] OR pancreatic surgery [tw] OR pancreatic resection [tw] OR distal pancreatectomy [tw] OR pancreatic left resection [tw] OR pancreaticoduodenectomy [tw]) AND (somatostatin [tw] OR octreotide [tw] OR vapreotide [tw] OR lanreotide [tw]) AND (randomized controlled trial [pt] OR controlled clinical trial [pt] OR randomized controlled trials [MESH] OR random allocation [MESH] OR double-blind method [MESH] OR single-blind method [MESH] OR clinical trial [pt] OR clinical trials [MESH] OR ("clinical trial" [tw]) OR ((singl* [tw] OR doubl* [tw] OR tripl* [tw]) AND (mask* [tw] OR blind* [tw])) OR placebos [MESH] OR placebo* [tw] OR random* [tw] OR research design [mh:noexp] OR Meta-Analysis [ptyp] OR systematic review [tw] NOT (animal [MESH] NOT human [MESH]) NOT case reports [pt] NOT Comment [PT] NOT letter [PT] NOT child [MESH] NOT infant [MESH])

#### Incision

(Surgery [MESH]) AND (midline incision [tw] OR transverse incision [tw] OR abdominal incision [tw] OR midline laparotomy [tw] OR transverse laparotomy [tw]) AND (randomized controlled trial [pt] OR controlled clinical trial [pt] OR randomized controlled trials [MESH] OR random allocation [MESH] OR double-blind method [MESH] OR single-blind method [MESH] OR clinical trial [pt] OR clinical trials [MESH] OR ("clinical trial" [tw]) OR ((singl* [tw] OR doubl* [tw] OR tripl* [tw]) AND (mask* [tw] OR blind* [tw])) OR placebos [MESH] OR placebo* [tw] OR random* [tw] OR research design [mh:noexp] OR Meta-Analysis [ptyp] OR systematic review [tw] NOT (animal [MESH] NOT human [MESH]) NOT case reports [pt] NOT Comment [PT] NOT letter [PT] NOT child [MESH] NOT infant [MESH])

#### Antibiotic prophylaxis

(Pancreas surgery [tw] OR pancreas resection [tw] OR pancreatic surgery [tw] OR pancreatic resection [tw] OR distal pancreatectomy [tw] OR pancreatic left resection [tw] OR pancreaticoduodenectomy [tw] OR hepatic surgery [tw] OR hepatic resection [tw] OR liver surgery [tw] OR hepatectomy [tw] OR colorectal surgery [tw] OR colon resection [tw] OR colon surgery [tw] OR abdominal surgery [tw] OR gastrointestinal surgery [tw]) AND (Antibiotic prophylaxis [tw] OR single shot [tw] OR antimicrobial prophylaxis [tw]) AND (randomized controlled trial [pt] OR controlled clinical trial [pt] OR randomized controlled trials [MESH] OR random allocation [MESH] OR double-blind method [MESH] OR single-blind method [MESH] OR clinical trial [pt] OR clinical trials [MESH] OR ("clinical trial" [tw]) OR ((singl* [tw] OR doubl* [tw] OR tripl* [tw]) AND (mask* [tw] OR blind* [tw])) OR placebos [MESH] OR placebo* [tw] OR random* [tw] OR research design [mh:noexp] OR Meta-Analysis [ptyp] OR systematic review [tw] NOT (animal [MESH] NOT human [MESH]) NOT case reports [pt] NOT Comment [PT] NOT letter [PT] NOT child [MESH] NOT infant [MESH] NOT child [MESH] NOT infant [MESH])

#### Nasogastric tube

(Pancreas surgery [tw] OR pancreas resection [tw] OR pancreatic surgery [tw] OR pancreatic resection [tw] OR distal pancreatectomy [tw] OR pancreatic left resection [tw] OR pancreaticoduodenectomy [tw] OR hepatic surgery [tw] OR hepatic resection [tw] OR liver surgery [tw] OR hepatectomy [tw] OR colorectal surgery [tw] OR colon resection [tw] OR colon surgery [tw] OR abdominal surgery [tw] OR gastrointestinal surgery [tw]) AND (Nasogastric decompression [tw] OR nasogastric tube [tw]) AND (randomized controlled trial [pt] OR controlled clinical trial [pt] OR randomized controlled trials [MESH] OR random allocation [MESH] OR double-blind method [MESH] OR single-blind method [MESH] OR clinical trial [pt] OR clinical trials [MESH] OR ("clinical trial" [tw]) OR ((singl* [tw] OR doubl* [tw] OR tripl* [tw]) AND (mask* [tw] OR blind* [tw])) OR placebos [MESH] OR placebo* [tw] OR random* [tw] OR research design [mh:noexp] OR Meta-Analysis [ptyp] OR systematic review [tw] NOT (animal [MESH] NOT human [MESH]) NOT case reports [pt] NOT Comment [PT] NOT letter [PT] NOT child [MESH] NOT infant [MESH] NOT child [MESH] NOT infant [MESH])

#### Abdominal drainage

(Surgery [MESH]) AND (abdominal drain* [tw] OR intraperitoneal drain* [tw] OR intraabdominal drain* [tw] OR prophylactic drain* [tw]) AND (randomized controlled trial [pt] OR controlled clinical trial [pt] OR randomized controlled trials [MESH] OR random allocation [MESH] OR double-blind method [MESH] OR single-blind method [MESH] OR clinical trial [pt] OR clinical trials [MESH] OR ("clinical trial" [tw]) OR ((singl* [tw] OR doubl* [tw] OR tripl* [tw]) AND (mask* [tw] OR blind* [tw])) OR placebos [MESH] OR placebo* [tw] OR random* [tw] OR research design [mh:noexp] OR Meta-Analysis [ptyp] OR systematic review [tw] NOT (animal [MESH] NOT human [MESH]) NOT case reports [pt] NOT Comment [PT] NOT letter [PT] NOT child [MESH] NOT infant [MESH] NOT child [MESH] NOT infant [MESH])

#### Postoperative feeding

(Pancreas surgery [tw] OR pancreas resection [tw] OR pancreatic surgery [tw] OR pancreatic resection [tw] OR distal pancreatectomy [tw] OR pancreatic left resection [tw] OR pancreaticoduodenectomy [tw] OR hepatic surgery [tw] OR hepatic resection [tw] OR liver surgery [tw] OR hepatectomy [tw] OR colorectal surgery [tw] OR colon resection [tw] OR colon surgery [tw] OR abdominal surgery [tw] OR gastrointestinal surgery [tw]) AND (feeding [tw] OR postoperative feeding [tw] OR enteral feeding [tw] OR nasogastric feeding [tw] OR nasojejunal feeding [tw] OR parenteral nutrition [tw] OR enteral nutrition [tw]) AND (randomized controlled trial [pt] OR controlled clinical trial [pt] OR randomized controlled trials [MESH] OR random allocation [MESH] OR double-blind method [MESH] OR single-blind method [MESH] OR clinical trial [pt] OR clinical trials [MESH] OR ("clinical trial" [tw]) OR ((singl* [tw] OR doubl* [tw] OR tripl* [tw]) AND (mask* [tw] OR blind* [tw])) OR placebos [MESH] OR placebo* [tw] OR random* [tw] OR research design [mh:noexp] OR Meta-Analysis [ptyp] OR systematic review [tw] NOT (animal [MESH] NOT human [MESH]) NOT case reports [pt] NOT Comment [PT] NOT letter [PT] NOT child [MESH] NOT infant [MESH] NOT child [MESH] NOT infant [MESH])

#### Patient mobilisation

(Pancreas surgery [tw] OR pancreas resection [tw] OR pancreatic surgery [tw] OR pancreatic resection [tw] OR distal pancreatectomy [tw] OR pancreatic left resection [tw] OR pancreaticoduodenectomy [tw] OR hepatic surgery [tw] OR hepatic resection [tw] OR liver surgery [tw] OR hepatectomy [tw] OR colorectal surgery [tw] OR colon resection [tw] OR colon surgery [tw] OR abdominal surgery [tw] OR gastrointestinal surgery [tw]) AND (mobilisation [tw] OR mobilization [tw] OR postoperative mobilisation [tw] OR patient mobilisation [tw] OR postoperative mobilization [tw] OR patient mobilization [tw]) AND (randomized controlled trial [pt] OR controlled clinical trial [pt] OR randomized controlled trials [MESH] OR random allocation [MESH] OR double-blind method [MESH] OR single-blind method [MESH] OR clinical trial [pt] OR clinical trials [MESH] OR ("clinical trial" [tw]) OR ((singl* [tw] OR doubl* [tw] OR tripl* [tw]) AND (mask* [tw] OR blind* [tw])) OR placebos [MESH] OR placebo* [tw] OR random* [tw] OR research design [mh:noexp] OR Meta-Analysis [ptyp] OR systematic review [tw] NOT (animal [MESH] NOT human [MESH]) NOT case reports [pt] NOT Comment [PT] NOT letter [PT] NOT child [MESH] NOT infant [MESH] NOT child [MESH] NOT infant [MESH])

## Supplementary Material

Additional file 1 **Survey on peri-operative standards in distal pancreatectomy**. A copy of the research instrument used for the survey. Click here for file

Additional file 2 **Meta-analyses and randomized controlled trials on perioperative management**. Appendix 1 summarizes relevant data from the meta-analyses and randomized controlled trials found by the literature search of best practice. Click here for file
