# Supplementary material for: Perioperative management in distal pancreatectomy: results of a survey in 23 European participating centres of the DISPACT trial and a review of literature
Source: Trials. 2009 Jul 26;10:58. doi: 10.1186/1745-6215-10-58 (PMC2726965; doi:10.1186/1745-6215-10-58)
Supplement: Additional file 1 — Survey on peri-operative standards in distal pancreatectomy. A copy of the research instrument used for the survey. [file 1745-6215-10-58-S1.pdf]

Survey on peri-operative standards in distal pancreatectomy

In order to evaluate peri-operative standards within the DISPACT-Trial group we kindly a to fill out this questionnaire according to the local standards at your institution.

1. Bowel preparation
   - ☐ No
   - ☐ Enema
   - ☐ Orthograde lavage

2. Thoracic epidural catheter (TEC)
   - ☐ No
   - ☐ Yes

   Medication of TEC
   - ☐ Local anaesthetics
   - ☐ Local anaesthetics and opioids

   Duration of TEC (days) |__|__|

3. Peri-operative sinlge-shot antibiotic prophylaxis
   - ☐ No
   - ☐ Yes

4. Type of incision
   - ☐ Midline
   - ☐ Transverse

5. Post-operative care
   - ☐ Intermediate care unit until day ______
   - ☐ Intensive care unit until day ______
   - ☐ Nursery ward

6. Post-operative gastric tube
   - ☐ No
   - ☐ Yes until day _______

7. Intraabdominal drain
   - ☐ No
   - ☐ Yes until day _______

8. Post-operative feeding
   - ☐ Intravenous until day ______
   - ☐ Via gastric tube beginning day ______
   - ☐ Via jejunal tube beginning day ______
   - ☐ Oral (see below for further details)

   Oral, beginning with fluids at
   - ☐ Day of surgery
   - ☐ 1sr postoperative day (pod)
   - ☐ 2nd pod
   - ☐ 3rd pod
   - ________pod

   Oral, beginning with solid food at
   - ☐ Day of surgery
   - ☐ 1sr postoperative day (pod)
   - ☐ 2nd pod
   - ☐ 3rd pod
   - ________pod

9. First mobilisation out of bed at
   - ☐ Day of surgery
   - ☐ 1sr postoperative day (pod)
   - ☐ 2nd pod
   - ☐ 3rd pod
   - ________pod

10. Peri-operative somatostatin therapy
    - ☐ No
    - ☐ Yes until day_______
