# Supplementary material for: Perioperative management in distal pancreatectomy: results of a survey in 23 European participating centres of the DISPACT trial and a review of literature
Source: Trials. 2009 Jul 26;10:58. doi: 10.1186/1745-6215-10-58 (PMC2726965; doi:10.1186/1745-6215-10-58)
Supplement: Additional file 2 — Meta-analyses and randomized controlled trials on perioperative management. Appendix 1 summarizes relevant data from the meta-analyses and randomized controlled trials found by the literature search of best practice. [file 1745-6215-10-58-S2.pdf]

Meta-analyses and randomized controlled trials on perioperative management

A. Bowel preparation	Title and author	Comment
Meta-analyses		
	Mechanical bowel preparation for elective colorectal surgery. Guenaga et al. 2005	9 trials / 1592 patients included. Bowel preparation is associated with increased rates of anastomotic leakage (6.3 vs. 3.2%; p=0.003) and wound complications (7.4 vs. 5.4, not significant).
	Meta-analysis of randomized clinical trials of colorectal surgery with or without mechanical bowel preparation. Slim et al. 2004	7 trials / 1454 patients included. Significantly more anastomotic leakage after mechanical bowel preparation (5.6 vs. 3.2%; p=0.032). No significant difference was found for wound infection, other septic complications and non-septic complications.
RCTs		
	Mechanical bowel preparation for elective colorectal surgery: a multicentre randomised trial. Contant et al. 2007	1431 patients / 13 centres included. No significant difference in anastomosic leakage, septic complications and mortality between bowel preparation and no bowel preparation but fewer abscesses after anastomotic leakage.
	Multicentre randomized clinical trial of mechanical bowel preparation in elective colonic resection. Jung et al. 2007	1505 patients enrolled. No significant differences in overall complications.
	Mechanical bowel preparation for elective colorectal surgery with primary intraperitoneal anastomosis by a single surgeon: interim analysis of a prospective single-blinded randomized trial. Pena-Soria et al. 2007	97 patients included. Same or worse outcomes after mechanical bowel preparation (Anastomotic failure: 8.3 vs. 4.1, not significant).
	Mechanical bowel preparation or not? Outcome of a multicenter, randomized trial in elective open colon surgery. Fa-Si-Oen et al. 2005	250 patients / centres included. No significant difference between groups.
	Randomized clinical trial of mechanical bowel preparation versus no preparation before elective left-sided colorectal surgery. Bucher et al. 2005	135 patients included. No bowel preparation is associated with reduced postoperative morbidity (22 vs. 8%; p=0.028).
	Is mechanical bowel preparation mandatory for elective colon surgery? A prospective randomized study. Ram et a. 2005	329 patients included. No advantage of bowel preparation in elective colorectal surgery.

B. Incision	Title and author	Comment
Meta-analyses		
	Transverse verses midline incisions for abdominal surgery. Brown et al. 2005	No difference in complication rates and recovery times.
RCTs		
	Randomized clinical trial of vertical or transverse laparotomy for abdominal aortic aneurysm repair. Fassiadis et al. 2005	69 patients included. Incisional hernia is less frequent after transverse incision.

C. Antibiotic prophylaxis	Title and author	Comment
Meta-analyses		
	Antimicrobial prophylaxis in colorectal surgery: a systematic review of randomized controlled trials. Song et al. 1998	147 trials included. Antibiotic prophylaxis prevents surgical wound infection after colorectal surgery. Single-shot is as effective as long-term postoperative antibiotic prophylaxis.
RCTs		
	Randomized, multicenter trial of antibiotic prophylaxis in elective colorectal surgery: single dose vs 3 doses of a second-generation cephalosporin without metronidazole and oral antibiotics. Fujita et al. 2007	384 patients / 7 centres included. Antibiotic dose correlates to incidence of incisional surgical site infections.

D. Abdominal drain	Title and author	Comment
Meta-analyses		
	Routine abdominal drainage for uncomplicated liver resection. Gurusamy et al. 2007	465 patients / 5 trials included. No significant difference between abdominal drainage vs. no drainage groups as far as mortality, intra-abdominal collections requiring re-operation, infected intra-abdominal collections, wound infection, ascitic leak, and hospital stay are concerned.
	Evidence-based value of prophylactic drainage in gastrointestinal surgery: a systematic review and meta-analyses. Petrowsky et al. 2004	30 trials included. Prophylactic drainage is indicated after esophageal resection and total gastrectomy, but not after hepatic, rectal or colonic resection with primary anastomosis and appendectomy.
RCTs		
	Prospective randomized clinical trial of the value of intraperitoneal drainage after pancreatic resection. Conlon 2001	179 patients (40 patients receiving distal pancreatectomy) included. No reduction in complication or mortality. No reduced need for interventional drainages and surgical exploration after septic complications.
	Randomized clinical trial of the effects of abdominal drainage after elective hepatectomy using the crushing clamp method. Sun et al. 2006	120 patients included. Cirrhosis and abdominal drainage independently correlate to development of postoperative wound complications. No difference in length of hospitalisation.

E. Pain management	Title and author	Comment
Meta-analyses		
	Meta-analysis of epidural analgesia versus parenteral opioid analgesia after colorectal surgery. Marret et al. 2007	16 trials included. No difference in length of hospitalisation.
	Patient controlled intravenous opioid analgesia versus continuous epidural analgesia for pain after intra-abdominal surgery. Werawatganon et al. 2005	711 patients / 9 studies included. Continuous epidural analgesia is superior in relieving postoperative pain but associated with a higher incidence of pruritus.
	Efficacy of postoperative patient-controlled and continuous infusion epidural analgesia versus intravenous patient-controlled analgesia with opioids: a meta-analysis. Wu et al. 2005	Continuous epidural infusion significantly is superior in relieving overall pain; higher incidence of nausea/vomiting and motor block but lower incidence of pruritus.
	Epidural local anaesthetics versus opioid-based analgesic regimens on postoperative gastrointestinal paralysis, PONV and pain after abdominal surgery. Jørgensen et al. 2000	Local anaesthetics decrease gastrointestinal paralaysis with comparable postoperative pain relief compared to regimens using opioids.
RCTs		
	Pain relief and safety after major surgery. A prospective study of epidural and intravenous analgesia in 2696 patients. Flisberg et al. 2003	2696 patients included. Less pain after epidural analgesia; opioid related side-effects more common with intravenous morphine analgesia.
	Comparison of intravenous or epidural patient-controlled analgesia in the elderly after major abdominal surgery. Mann et al. 2000	70 patients included. Epidural patient controlled anaesthesia using local anaesthetics and an opioid provides better pain relief and improves mental status and bowel activity compared to intravenous application.

F. Gastric tube	Title and author	Comment
Meta-analyses		
	Prophylactic nasogastric decompression after abdominal surgery. Nelson et al. 2007	5240 patients / 33 trials included. Earlier return of bowel function, decreased pulmonary complications and shorter hospitalisation without gastric tube.
	Systematic review of prophylactic nasogastric decompression after abdominal operations. Nelson et al. 2005	4194 patients / 28 trials included. Earlier return of bowel function without gastric tube.
RCTs		
	Randomized clinical trial evaluating the need for routine nasogastric decompression after elective hepatic resection. Pesseaux et al. 2007	200 patients included. No advantage but increased risk of pulmonary complications in nasogastric tube group.

G. Postoperative feeding	Title and author	Comment
Meta-analyses		
	Early enteral nutrition within 24h of colorectal surgery versus later commencement of feeding for postoperative complications. Andersen et al. 2006	1173 patients / 13 trials included. No significant advantage in keeping patients starved after gastrointestinal surgery.
RCTs		
	Randomized clinical trial of the impact of early enteral feeding on postoperative ileus and recovery. Han-Geurts et al. 2007	128 patients included. No significant difference between early (median 2) vs. conventional (median 5) return to oral diet.

H. Patient mobilisation	Title and author	Comment
RCTs		
	The quantity of early upright mobilisation performed following upper abdominal surgery is low: an observational study. Browning et al. 2007	50 patients included. Increased early upright mobilisation may have positive effect on reduced length of hospitalisation.
	Randomised clinical trial of physiotherapy after open abdominal surgery in high risk patients. Mackay et al. 2005	56 patients included. In high risk patients, deep breathing and coughing exercises do not significantly decrease postoperative pulmonary complications.
	Randomized controlled trial of prophylactic chest physiotherapy in major abdominal surgery. Fagevik et al. 1997	366 patients included. Preoperative chest physiotherapy significantly decreased postoperative pulmonary complications and improved mobilisation.

I. Somatostatin	Title and author	Comment
Meta-analyses		
	Use of octreotide for the prevention of pancreatic fistula after elective pancreatic surgery: a systematic review and meta-analysis. Alghamdi et al. 2007	1359 patients / 7 trials included. Somatostatin significantly reduces incidence of pancreatic fistula after elective pancreatic surgery. No significant difference in mortality rates.
	Efficacy of somatostatin and its analogues in prevention of postoperative complications after pancreaticoduodenectomy: a meta-analysis of randomized controlled trials. Zeng et al. 2008	8 studies included. Somatostatin does not significantly reduce postoperative complications after pancreaticoduodenectomy.
	Meta-analysis of the value of somatostatin and its analogues in reducing complications associated with pancreatic surgery. Connor et al. 2005	1918 patients / 10 trials included. Somatostatin reduces postoperative complications but not mortality.
RCTs		
	Prospectively randomized trial using perioperative low-dose octreotide to prevent organ-related and general complications after pancreatic surgery and pancreatico-jejunostomy. Hesse et al. 2005	105 patients included. No significant difference in occurrence of pancreatic fistula and overall morbidity and mortality in somatostatin vs. no somatostatin group.
	Effects of somatostatin prophylaxis after pylorus-preserving pancreaticoduodenectomy: increased delayed gastric emptying and reduced plasma motilin. Shan et al. 2005	23 patients included. Delayed gastric emptying more frequent after somatostatin prophylaxis.
	Somatostatin analogues in the prevention of pancreas-related complications after pancreatic resection. Ramos-De la Medina et al. 2006	381 patients included. No benefit of perioperative somatostatin administration.
